# Lymph node yield as a surrogate marker for tumour biology and prognosis in colon cancer

**DOI:** 10.1038/s41416-025-02949-y

**Published:** 2025-02-14

**Authors:** James Bundred, Nikhil Lal, Dedrick K. H. Chan, Simon J. A. Buczacki

**Affiliations:** 1 Nuffield Department of Surgical Sciences, University of Oxford, Oxford, UK; 2 NUS Centre for Cancer Research, Yong Loo Lin School of Medicine, Singapore, Singapore

**Keywords:** Colon cancer, Surgical oncology

## Abstract

**Background:**

We interrogated two large national databases to explore the underlying mechanisms and institutional effects of the known association of enhanced survival with a higher lymph node yield (LNY) in non-metastatic colon cancer.

**Method:**

Clinical and pathological data for stage I-III colon adenocarcinomas were extracted from the CORECT-R (England, 2010–2020) and SEER database (USA, 2000–2020). A lymph node (LN) cut-off for the lack of clinically significant increase in nodal positivity was identified. A multivariable Cox-regression model was developed to study the effect of LNY on overall survival. Furthermore, institutional variations in LNY and their impact on survival were explored.

**Results:**

Patients were retrospectively included from the CORECT-R (*n* = 84,116) and SEER (*n* = 287,974) databases. No significant increase in nodal positivity was noted after a LN cut-off of 9. However, improved survival was noted in node-negative and node-positive cancers beyond this cut-off. A 1% risk-reduction concerning overall survival was reported for every node counted. We identified ten outlying institutions across England with an observed LNY greater or less than the expected, with no impact on overall survival.

**Discussions:**

We advocate incorporating LNY into patient and clinician discussions as a surrogate marker of tumour biology and prognosis rather than using LNY as a quality indicator.

## Introduction

Colon cancer is the fourth most common cancer worldwide and accounts for over 5% of all cancer mortality therefore representing a significant burden on healthcare systems globally [1]. Surgical resection of the primary tumour and surrounding lymph nodes (LN) forms the mainstay of curative treatment for the most colon cancer patients. The LN yield (LNY) and number of metastatic nodes are of interest as both prognostic and surgical quality indicators. LN metastasis, irrespective of other tumour characteristics, is regarded as a critical prognostic factor and signifies the need for adjuvant chemotherapy [2]. Several studies have demonstrated a survival advantage in patients with a higher LNY in node-negative and node-positive colon cancer [3–5].

Substantial work has aimed to establish the role of lymphadenectomy in curative cancer surgery. Dating back to the 19th century, William Halsted proposed that breast cancer was a local disease and progressed in a predictable stepwise manner from the primary tumour to regional LNs and subsequently to distant organs [6]. He thereby advocated radical mastectomies with extended lymphadenectomy as a curative procedure. In contrast, Bernard Fisher and Blake Cady proposed that breast cancer is a systemic disease with a haematogenous spread of malignant cells to organs occurring early and recommending lymphadenectomy for staging only [7].

In colon cancer, stage migration has gained prominence as an explanation for enhanced survival in patients with a higher LNY [8]. Stage migration survival effects can be explained by both Halstedian and Cady-Fisher mechanisms. The survival advantage of an increased LNY is proposed to be due to the identification of a greater number of positive LNs and, thereby, ‘correctly’ staging such patients, resulting in higher chances of obtaining adjuvant therapy [9]. However, stage migration effects have been challenged in several observational studies where no significant change in the number of positive LNs with increased LNs harvested was found [3, 5, 10]. An alternative explanation of better tumour biology and tumour-host interaction resulting in an enhanced immune response in higher LNY patients has also been suggested [4, 11]. No study has yet disentangled the relative survival effects of stage migration and tumour biology on colon cancer.

The optimal LNY in colon cancer resections is controversial and has been debated for over three decades [12]. Current guidelines recommend 12 as the minimal cut-off for adequate staging, a figure dating back to the World Congress of Gastroenterology recommendations in 1990 [13]. Since then, several international guidelines have endorsed 12 as an optimal LN cut-off [14]. However, based on scientific evidence, there appears to be no consensus on an optimal cut-off, as fluctuating LN cut-off ranging between 6 and 40 has been reported [10]. In routine clinical practice, LNY is multi-factorial and depends on the surgeon, pathologist, institution and patient-related factors [15]. A cut-off of 12 has been proposed as a quality indicator, and extensive observational studies across the United States of America have demonstrated that this cut-off is only satisfied in under 50% of hospitals [16–18], with superior compliance seen at academic and high-volume centres [19]. Currently, there are no studies that have evaluated LNY as a quality indicator within colon cancer multi-disciplinary teams (MDT) across England despite significant variations in yields between hospitals having previously been uncovered in a small regional study [20]. The need for a broader analysis of LNY across England is augmented by a recent study showing worse colon cancer survival rates in England compared to other European countries [21].

This study interrogates two large national databases of colon cancer patients in England and the USA to explore the underlying mechanisms and institution-driven effects of the known association of enhanced survival with LNY in non-metastatic colon cancer.

## Methods

### Study cohorts

The COloRECTal cancer data Repository (CORECT-R) was analysed to identify patients with stage I-III colon adenocarcinoma who underwent curative surgery in England between 2010 and 2020 [22]. To provide a validation dataset, the Surveillance, epidemiology and end results (SEER) database from the USA was also used to identify all patients between 2000 and 2020 with site code ICD-0-3, colon excluding rectum [23]. Only patients with non-metastatic adenocarcinoma of the colon and complete LN data were included. Patients undergoing emergent procedures, subtotal colectomies, pan-proctocolectomies or exenterations were excluded across both datasets. The study protocol was approved by the Hub Access Committee of the UK Colorectal Cancer Intelligence Hub and ethical approval was given by South West Central Bristol Research Ethics Committee 18/SW/0134. All methods described were performed in accordance with relevant guidelines and regulations.

### Data collection

Both datasets were explored for variables such as age, sex, tumour site, LNY, number of positive LNs, procedure performed, pathological stage, adjuvant treatment and overall survival. Additionally, Charleston co-morbidity index, frailty and deprivation decile were included for the CORECT-R dataset. Survival was calculated in years, from the date of diagnosis to the date of death or censoring (31st December 2020).

### Statistical analysis

Demographic data are presented as median and inter-quartile range. *P*-values are from chi-squared analysis for categorical variables and f-test for continuous variables where a normal distribution was demonstrated. The percentage of patients with at least one LN containing metastasis for every LNY was calculated and plotted to identify a value at which an increase in LNY failed to improve detection of patients with LN-positive disease. Associations between LNY and overall survival were examined using conventional Cox-proportional hazards modelling and parametric survival modelling to allow assessment of the linearity of the association between the increase in LNY and increasing survival [24]. A log-normal parametric survival model was chosen after assessment of log-normal, exponential and Weibull models (by Akiake information criterion and Bayesian information criterion) and comparison of all models to Cox-proportional hazards models to assess fit [25]. As the number of events compared to variables in our datasets were non-restrictive, all variables associated with survival were included in multivariable modelling where appropriate and co-linearity was checked by calculation of variance inflation factors. All statistical analysis was completed using R version 4.0 (R Foundation for Statistical Computing, Vienna, Austria) [26].

#### Ethics approval and consent to participate

The project is covered by the Establishing a UK Colorectal Cancer Intelligence Hub Research Ethics approval (23/SW/0069) granted by the South West – Central Bristol Research Ethics Committee. As this was a retrospective, anonymised analysis of patient data, explicit consent for this study was waived.

## Results

### Patient cohorts

**Fig. 1:**
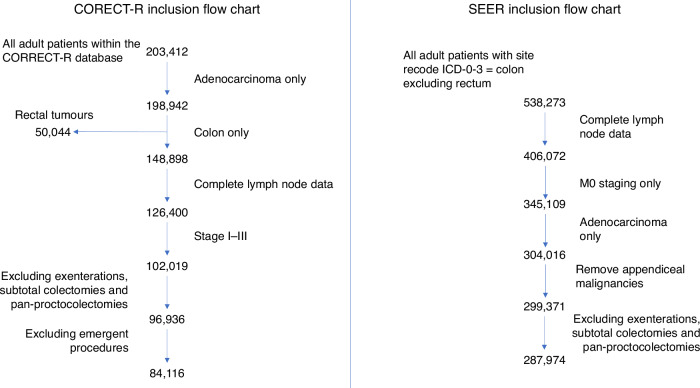
Consort diagram of inclusion and exclusion criteria for both CORECT-R and SEER cohorts.

As per inclusion criteria, 84,116 and 287,974 patients were included from the CORECT-R and SEER databases from England and the USA, respectively (Fig. 1).

The median LNY was 18.0 and 18.1, and the median (inter-quartile range) number of positive LNs was 0 (0–2) and 0 (0–2), respectively, from the CORECT-R and SEER cohorts (Table 1). The median length of follow-up was 42 months (19–70) and 60 months (24–118), respectively.

**Table 1:** Demographics of the CORECT-R and SEER cohorts.

		CORECT-R			SEER		
		<9	>=9	*p*	<9	>=9	*p*
Age	Median (IQR)	73.0 (66.0 to 80.0)	72.0 (64.0 to 79.0)	<0.001	72.0 (63.0 to 80.0)	70.0 (60.0 to 79.0)	<0.001
Sex	Male	3368 (56.0)	40,771 (52.2)	<0.001	24,771 (51.7)	117,004 (48.8)	<0.001
	Female	2645 (44.0)	37,332 (47.8)		23,186 (48.3)	122,962 (51.2)	
Deprivation decile	1 - least deprived	1319 (21.9)	18,168 (23.3)	0.024			
	2	1531 (25.5)	18,589 (23.8)				
	3	1229 (20.4)	16,306 (20.9)				
	4	1066 (17.7)	13,809 (17.7)				
	5 - most deprived	868 (14.4)	11,231 (14.4)				
Site	Right colon	2402 (39.9)	47,011 (60.2)	<0.001			
	Left colon	3611 (60.1)	31,092 (39.8)				
Number of positive nodes	Median (IQR)	0.0 (0.0 to 1.0)	0.0 (0.0 to 2.0)	<0.001	0.0 (0.0 to 0.0)	0.0 (0.0 to 1.0)	<0.001
Total nodes excised	Median (IQR)	8.0 (6.0 to 9.0)	19.0 (15.0 to 25.0)	<0.001	6.0 (4.0 to 7.0)	17.0 (13.0 to 23.0)	<0.001
Charlson comorbidity index	0	4189 (69.7)	57,464 (73.6)	<0.001			
	1	1097 (18.2)	12,900 (16.5)				
	2	392 (6.5)	4582 (5.9)				
	3	335 (5.6)	3157 (4.0)				
Scarf Frailty Score	Fit	3259 (54.2)	44,072 (56.4)	<0.001			
	Mild frailty	1336 (22.2)	17,422 (22.3)				
	Moderate frailty	773 (12.9)	9566 (12.2)				
	Severe frailty	645 (10.7)	7043 (9.0)				
Overall Stage	Unknown	1014 (16.9)	8511 (10.9)	<0.001			
	0	0 (0.0)	0 (0.0)				
	1	1577 (26.2)	11,063 (14.2)				
	2	1869 (31.1)	31,730 (40.6)				
	3	1553 (25.8)	26,799 (34.3)				
Pathological T Stage	Unknown T stage	90 (1.5)	710 (0.9)	<0.001			
	T0	3 (0.0)	9 (0.0)				
	T1	1015 (16.9)	4341 (5.6)				
	T2	1222 (20.3)	10,350 (13.3)				
	T3	2528 (42.0)	44,702 (57.2)				
	T4	1155 (19.2)	17,991 (23.0)				
Pathological N Stage	Unknown N stage	146 (2.4)	1416 (1.8)	<0.001			
	N0	4016 (66.8)	47,137 (60.4)				
	N1	1528 (25.4)	19,258 (24.7)				
	N2	322 (5.4)	10,282 (13.2)				
	N3	1 (0.0)	10 (0.0)				
Adjuvant Chemotherapy	unknown	3031 (50.4)	27,196 (34.8)	<0.001			
	Adjuvant chemotherapy	540 (9.0)	13,605 (17.4)		8630 (18.0)	67,038 (27.9)	<0.001
	No adjuvant chemotherapy	2442 (40.6)	37,302 (47.8)		39,327 (82.0)	172,928 (72.1)	<0.001

### Change in LNY and nodal positivity rates over time

**Fig. 2:**
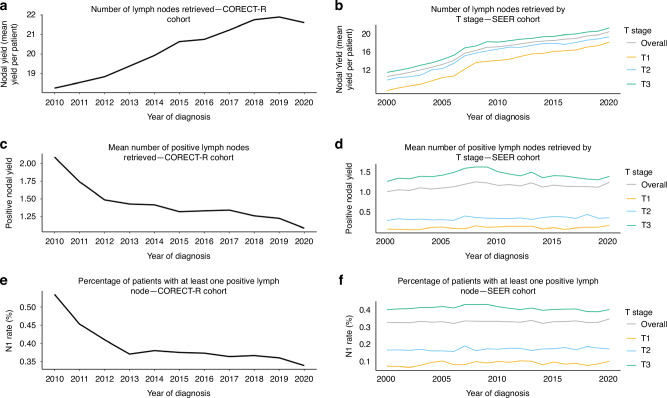
Lymph node metrics over time for the CORECT-R and SEER cohorts. Line graphs showing **a** the number of lymph nodes removed per patient on average for each year included in the CORECT-R database **b** the same for the SEER database **c** the number of lymph nodes containing cancer removed per patient on average across the CORECT-R database **d** the same for the SEER database **e** the percentage of patients with at least one lymph node metastasis per year in the CORECT-R database **f** the percentage of patients with at least one lymph node metastasis per year in the SEER database.

The LNY steadily increased from 2010 to 2020 across all stages in the CORECT-R cohort and all T stages in the SEER cohort (Fig. 2a, b). Across the same time period, the average number of positive nodes per patient decreased across all cancer stages in the CORECT-R cohort and across T3 patients in the SEER cohort (Fig. 2c, d). As a result, the overall LN positivity rate declined from over 50% in 2010 to below 40% in the CORECT-R cohort. In the SEER cohort, the nodal positivity rate declined in T3 patients over the same period (Fig. 2e, f).

### The impact of increasing LNY on nodal positivity rates

**Fig. 3:**
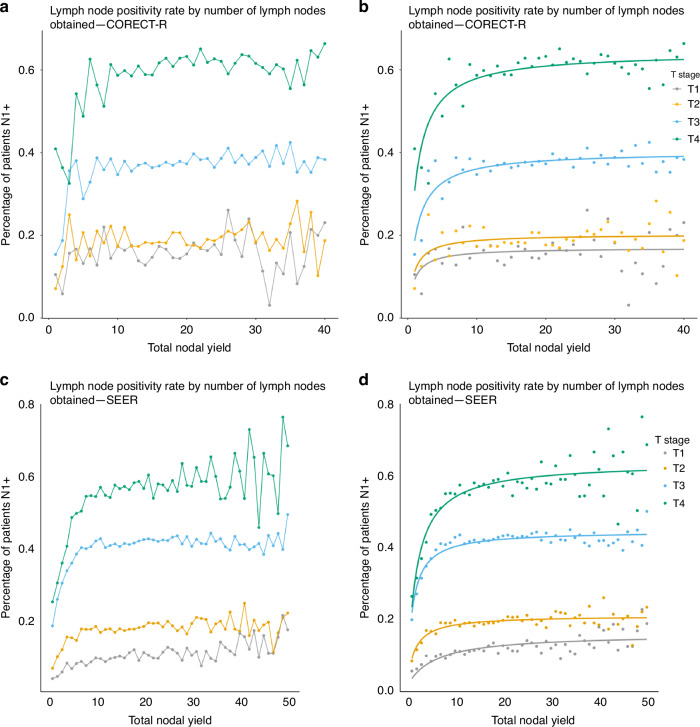
The percentage of patients with at least one lymph node metastasis at every value of lymph node yield. **a** For CORECT-R patients and with non-linear regression applied to show approximate trend (**b**). **c** For SEER patients and with non-linear regression applied to show approximate trend (**d**).

For both datasets LN positivity rate (N+ rate) increased up to a count of 9 LNs, at which point increasing the LNY had minimal impact on LN positivity rates (Fig. 3a–d). Above an LNY of 9, the increase in LN positivity rate with each increasing LN identified was 0.11% for T3 tumours, 0.06% for T2 tumours and 0.23% for T1 tumours in the CORECT-R dataset. Above an LNY of 9, the increase in LN positivity with each increasing LN identified was 0.07% for T3 tumours, 0.05% for T2 tumours and 0.1% for T1 tumours in the SEER dataset.

### The impact of increasing LNY on overall survival

**Fig. 4:**
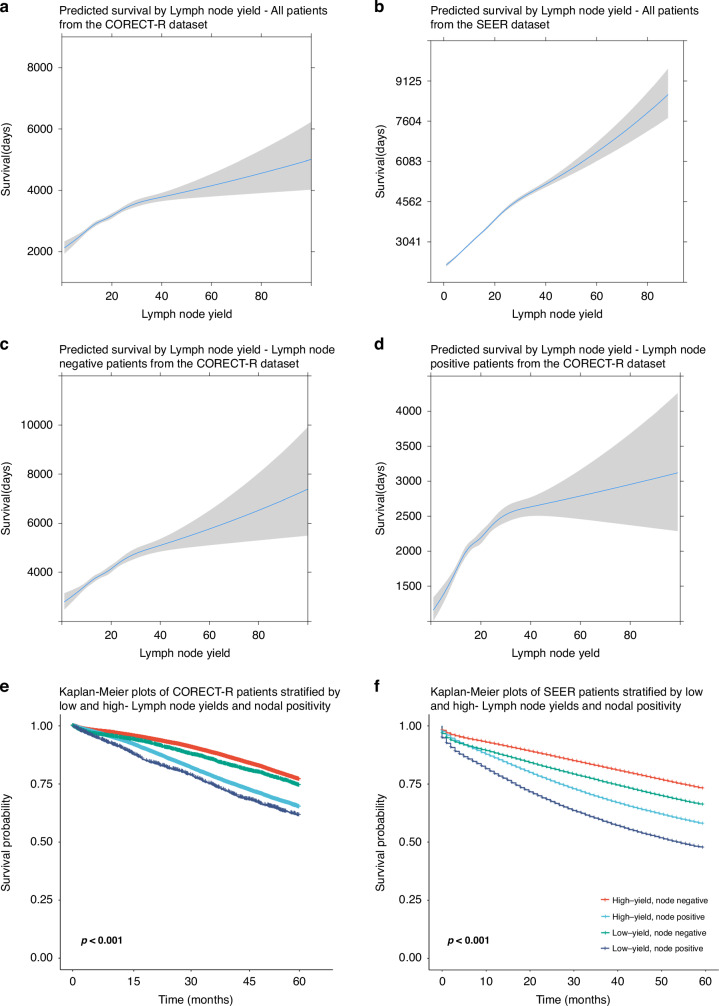
Survival modelling by different lymph node yields and nodal status. Predicted survival times for each lymph node yield were then plotted based on the parametric survival model created and 95% confidence intervals included in grey **a** CORECT-R cohort overall model **b** SEER cohort overall model **c** CORECT-R N0 patients only **d** CORECT-R patients with at least one lymph node metastasis only. Kaplan Meier plots of patients cross-stratified by yield (>9 vs ≤9) and nodal positivity (N+ vs N0) for **e** CORECT-R. **f** SEER for up to 60 months follow up following resection.

A lognormal parametric survival model was fit to examine the correlation between LNY and survival (Fig. 4a–d). Increasing LNY was associated with a survival time ratio of 1.01(1.01–1.01). There was a linear increase in the predicted overall survival between LN counts ranging from 1 to 30 for both CORECT-R and SEER datasets.

Using Cox-proportional hazards modelling, increasing LNY was associated with increased overall survival (CORECT-R: HR per LN increase:0.99, 95% CI: 0.99–1.00, *p* < 0.001, SEER: HR per LN increase:0.99, 95% CI: 0.99–0.99, *p* < 0.001), when adjusting for other factors associated with improved survival (age, sex, deprivation, co-morbidity, number of nodes containing tumour, receipt of adjuvant chemotherapy) (Supplementary Table 1). In SEER data, increasing LNY was associated with a survival time ratio of 1.019(1.018–1.024). Cox-proportional hazards modelling found increasing LNY was associated with increased overall survival (HR per LN increase:0.99, 95% CI: 0.99–0.99, p < 0.001), when adjusting for other factors associated with improved survival (age, sex, year of surgery, race, site of tumour, number of nodes containing tumour, receipt of adjuvant chemotherapy and receipt of adjuvant radiotherapy) (Supplementary Table 1).

### The relative impacts of LNY and nodal positivity on overall survival

A subgroup analysis was performed to evaluate the impact of LNY separately in both patients with and without LN metastases. Multivariable Cox regression modelling was used to examine the relative impacts of total LNY and the number of positive LNs on overall survival. In the CORECT-R dataset, each additional LN was associated with an HR of 0.98 (95% CI 0.98–0.98, *p* < 0.001) and each individual LN containing tumour was associated with an HR of 1.07 (1.06–1.07, *p* < 0.001). (Supplementary Table 2). In the SEER dataset, each individual additional LN was associated with an HR of 0.98 (95% CI 0.98–0.98, *p* < 0.001) and each individual LN containing tumour was associated with an HR of 1.08 (1.08–1.08, *p* < 0.001) (Supplementary Table 2).

When segregating patients by a LNY cut-off of 9 and nodal positivity both low LNY patients and node positive patients had worse outcomes than their comparators on both CORECT-R and SEER datasets (Fig. 4e, f).

### Variation in LN retrieval across England

**Fig. 5:**
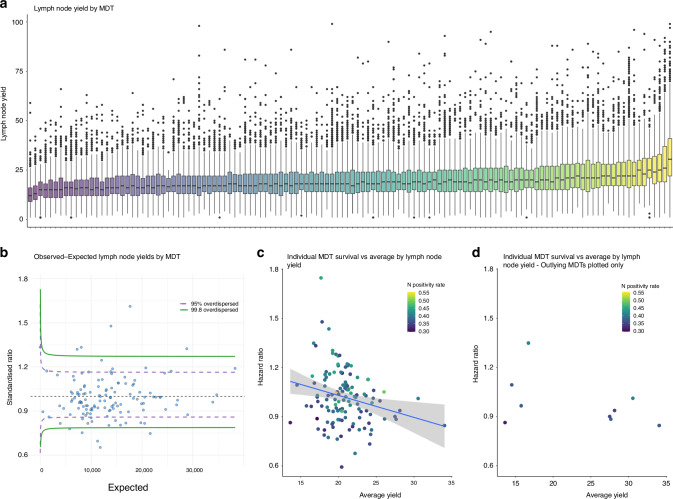
Examination of variation in lymph node yields across MDTs. **a** Boxplot of nodal yields by MDT. Interquartile range, median and outliers are plotted for each MDT. Anonymised MDTs are ordered by median nodal yield. **b** Funnel plot of observed vs expected lymph node yields by MDT with 95% and 99.8% dispersion lines to identify outlying MDTs. **c** Survival by lymph node yield at the MDT level re-capitulates individual patient data. Each dot represents an individual MDT. Hazard ratios are plotted as the individual MDT compared to all other MDTs. Dots are coloured by nodal positivity rate. Trend displayed in Fig. 5 did not reach statistical significance (*p* > 0.05). **d** Survival by lymph node yield and dots colour coded for nodal positivity rate for outlying trusts identified in (**b**).

Variation in LNY across the CORECT-R dataset for every MDT was individually examined (Fig. 5a). A linear regression model was developed to predict the expected number of LNs per patient. Observed versus expected LNs were then plotted for every MDT to identify outlying MDTs (Fig. 5b). An MDT was classed as an outlier if the observed/expected LN ratio was over two standard deviations from its mean. We identified ten outlying MDTs with an observed LNY greater or lower than the expected LNY. There were no significant variations in the overall survival of patients in the outlying MDTs in comparison to non-outlying MDTs (Fig. 5c, d).

## Discussion

With over 370,000 patients from two large databases across England and the USA, this is the largest study to report on the relative influence of LNY and tumour biology on colon cancer survival. We found a significant improvement in survival outcomes with an increase in LNY [3, 4], importantly, in both node-positive and node-negative colon cancers. The underlying reason for this improved survival has been debated for the last three decades. Two contrasting theories of stage migration and tumour-host immune response have been described as the reason for the improved survival with an increased LNY.

Our results additionally find no significant increase in nodal positivity after a cut-off of 9 LNs. This cut-off of 9 is maintained across all T-stages of colon cancer in the SEER and CORECT-R datasets. Additional LNs resected or identified in the specimen after this cut-off were very unlikely to change the staging of the colon cancer. However, despite a lack of stage migration effects, the survival outcomes continued to improve in node-negative and node-positive cases with increasing LNY. Based on our previous work, any survival benefit noted in patients with a raised LNY is, therefore, likely due to a robust anti-tumour immune response leading to more LNs being identified in the colon cancer specimen [4, 11]. Substantial evidence now highlights an increase in immune cell infiltration in colon cancers with a higher LNY. Multiple recent studies have identified increasing infiltration of T-cells within colon tumours with an increased lymph node yield [11, 27, 28]. Our recent work analysing multiomic data from The Cancer Genome Atlas demonstrated that adaptive and innate immune transcriptional changes in colon cancers were directly associated with higher LNYs irrespective of nodal status [4].

Other studies have attempted to identify a cut-off for stage migration in colon cancers. Baxter et al. analysed over 100,000 pT3 colon cancers noting only a marginal increase in nodal positivity after a cut-off of 6 [29]. We predict the lower cut-off for nodal positivity changes identified by Baxter and colleagues is related to the lower median LNY of 11 in their dataset compared to the median LNY of 18 in this study. Indeed, only 55% of their patients had an LNY of 12 or above. Our study included all T-stages applying adjustment to ensure a broader understanding regarding a cut-off could be obtained.

Based on apparent oncological advantages, surgeons have attempted to increase their LNYs by advocating radical lymphadenectomies. The Japanese Research Society for Cancer of the Colon and Rectum has recommended D3 lymphadenectomy for all colon cancers invading the muscularis mucosa (T2 or above) [30]. Meanwhile, in Europe, Hohenberger and colleagues in 2009 described complete mesocolic excision (CME) with central vessel ligation, an ideological translation of total mesorectal excision [31]. The group demonstrated that the quality of CME was associated with long-term survival outcomes [31]. However, a consensus on the impact of CME on survival outcomes has yet to be reached. RELARC trial has demonstrated an increase in median LNY with CME compared to non-CME (26 vs 23) [32]. However, this did not translate into an increase in nodal positivity. Similarly, the Italian CoME-in trial noted an increase in LNY with CME in comparison to non-CME resection with no difference in nodal staging [33]. The current study is not equipped to assess the survival outcomes with CME and D3 lymphadenectomy. However, evaluating the survival outcomes with extended lymphadenectomy to increase the LNY can be assessed in our data. Taken in the context of minimal up-staging with LNY > 9, one can only conclude that increasing LNY by extended lymphadenectomy is ineffective in improving survival outcomes but may increase morbidity.

Currently, there is no consensus on the role of extended lymphadenectomy for colon cancers clinically staged as node-positive before resection. In this scenario, surgeons may attempt a radical resection to eliminate the nodal pathway of metastasis. However, this is the first study to highlight that in node-positive disease, there is an insignificant influence of a Halstedian extended lymphadenectomy on nodal positivity and overall survival. Therefore, these findings are pertinent to surgeons undertaking radical resections for radiologically node-positive tumours to facilitate an evidence-based discussion with the patients regarding the risks and benefits of an extended lymphadenectomy.

In 2023, the Royal College of Pathologists (RCPath) in the United Kingdom (UK) released the ‘Dataset for Histopathological Reporting of Colorectal Cancer’ [34]. The guidance recommends raising the minimum median LNs examined per case from 12 to 15 to increase the probability of detecting LN metastasis. Furthermore, the document recommends an audit of the median LNs examined per case for use as a quality indicator [34]. Our results do not support raising the minimum LN count to detect positive LNs per se, as there appears to be minimal gain in nodal positivity after a count of 9. LNY is multi-factorial and dependent on variables relating to patients and pathologist [35]. Perhaps more importantly, any LN cut-off does not account for the underlying tumour biology resulting in the raised LNY. Consequently, alterations to surgical and pathology practices to increase the LNY are unlikely to benefit the treatment and will mislead if used as a quality indicator. We agree however with the RCPath recommendation that pathologists should count *all* LNs in the specimen. The objective being to identify patients with a more or less robust anti-tumour immune response rather than attempting to identify larger numbers of nodal metastases. This recommendation is based on the evolution in our understanding of immune contexture and the combined use of LNY and lymphocyte infiltration to predict prognosis by identifying immune-competent tumours [36, 37]. In circumstances where a cut-off for adequate staging has not been achieved, the authors advocate these tumours to be treated as “high-risk” colon cancers and be offered adjuvant chemotherapy to counter the combined effects of under-staging and poor tumour biology [38].

This is the first study to report LNY and overall survival across 136 MDTs across England. This national evaluation emphasises that MDTs adequately stage colon cancers in England. Despite adequate staging, there is widespread variation in nodal positivity across the MDTs. Our study is limited by being retrospective in design, however, a large sample size across two Western healthcare systems would be challenging to achieve prospectively. We did not include patients with stage IV colon cancers and rectal cancers due to the vast majority of these patients having neoadjuvant treatment and its known effect on LNY [39], however, this study population is otherwise generalisable to a western population with colon cancer.

## Conclusion

Overall, this is the most extensive study to date to identify a cut-off for stage migration and quantify the dominant role of immune response in patients with a higher LNY and better survival. The study challenges the role of extended lymphadenectomy in colon cancer resections and the use of LNY as a quality indicator. The results will inform policymakers on standardising surgical and pathological factors to ensure adequate staging. Furthermore, we advocate incorporating LNY into patient and clinician discussions as a surrogate marker of tumour biology and prognosis. Ultimately, we anticipate this will enhance patients’ understanding of their prognosis and augment the delivery of personalised treatment for colon cancers.

## Supplementary information

Supplementary Tables

## Data Availability

Data available within the article or its supplementary materials.

## Code availability

The code used to analyse data and generate figures included within this manuscript is available upon reasonable request to the corresponding author.
