# Supplementary material for: Lymph node yield as a surrogate marker for tumour biology and prognosis in colon cancer
Source: Br J Cancer. 2025 Feb 14;132(7):643–51. doi: 10.1038/s41416-025-02949-y (PMC11961567; doi:10.1038/s41416-025-02949-y)
Supplement: Supplementary file 1 — Supplementary Tables [file 41416_2025_2949_MOESM1_ESM.docx]

|  |  | CORECT-R |  | SEER |  |
| --- | --- | --- | --- | --- | --- |
|  |  | HR (univariable) | HR (multivariable) | HR (univariable) | HR (multivariable) |
| Total nodes excised | Mean (SD) | 0.99 (0.99-1.00, p<0.001) | 0.99 (0.99-0.99, p<0.001) | 0.99 (0.99-0.99, p<0.001) | 0.99 (0.99-0.99, p<0.001) |
| Age | Mean (SD) | 1.05 (1.05-1.05, p<0.001) | 1.05 (1.05-1.05, p<0.001) | 1.06 (1.06-1.06, p<0.001) | 1.06 (1.06-1.06, p<0.001) |
| Sex | Male | - | - | 1.05 (1.04-1.06, p<0.001) | 1.22 (1.20-1.23, p<0.001) |
|  | Female | 0.89 (0.87-0.91, p<0.001) | 0.85 (0.83-0.87, p<0.001) | - | - |
| Deprivation decile | 1 - least deprived | - | - | - | - |
|  | 2 | 1.06 (1.03-1.10, p=0.001) | 1.06 (1.02-1.09, p=0.001) | - | - |
|  | 3 | 1.12 (1.08-1.16, p<0.001) | 1.12 (1.08-1.16, p<0.001) | - | - |
|  | 4 | 1.17 (1.13-1.22, p<0.001) | 1.17 (1.12-1.21, p<0.001) | - | - |
|  | 5 - most deprived | 1.30 (1.25-1.34, p<0.001) | 1.31 (1.26-1.36, p<0.001) | - | - |
| Charlson Comorbidity index | 0 | - | - | - | - |
|  | 1 | 1.56 (1.52-1.61, p<0.001) | 1.12 (1.09-1.16, p<0.001) | - | - |
|  | 2 | 2.08 (1.99-2.16, p<0.001) | 1.18 (1.12-1.23, p<0.001) | - | - |
|  | 3 | 3.06 (2.93-3.20, p<0.001) | 1.36 (1.29-1.43, p<0.001) | - | - |
| Scarf Frailty Score | Fit | - | - | - | - |
|  | Mild frailty | 1.57 (1.53-1.62, p<0.001) | 1.25 (1.21-1.29, p<0.001) | - | - |
|  | Moderate frailty | 2.16 (2.09-2.23, p<0.001) | 1.46 (1.41-1.52, p<0.001) | - | - |
|  | Severe frailty | 3.26 (3.15-3.37, p<0.001) | 1.93 (1.85-2.02, p<0.001) | - | - |
| Site of tumour | Right colon | - | - | -- | - |
|  | Transverse and left colon | 0.76 (0.74-0.78, p<0.001) | 0.92 (0.90-0.94, p<0.001) | - | - |
| Number of positive nodes | Mean (SD) | 1.08 (1.08-1.08, p<0.001) | 1.10 (1.10-1.10, p<0.001) | 1.07 (1.07-1.07, p<0.001) | 1.10 (1.10-1.10, p<0.001) |
| Adjuvant Chemotherapy | unknown | - | - | - | - |
|  | Adjuvant chemotherapy | 1.48 (1.42-1.54, p<0.001) | 1.73 (1.66-1.81, p<0.001) | 0.73 (0.72-0.74, p<0.001) | 0.88 (0.87-0.90, p<0.001) |
|  | No adjuvant chemotherapy | 1.78 (1.73-1.83, p<0.001) | 1.62 (1.58-1.67, p<0.001) | - | - |

Supplementary table 1 – Cox proportional hazards modelling of overall survival for both the CORECT-R and SEER cohorts

|  |  | all | HR (univariable) | HR (multivariable) |
| --- | --- | --- | --- | --- |
| Age | Mean (SD) | 71.2(11.3) | 1.06(1.06-1.06, p<0.001) | - |
| Sex | Male | 27490(53.2) | - | - |
|  | Female | 24208(46.2) | 0.83(0.80-0.85, p<0.001) | - |
| Deprivation decile | 1 - least deprived | 12054(23.3) | - | - |
|  | 2 | 12443(24.1) | 1.04(1.00-1.09, p=0.069) | - |
|  | 3 | 10753(20.8) | 1.09(1.04-1.14, p<0.001) | - |
|  | 4 | 9081(17.6) | 1.18(1.12-1.23, p<0.001) | - |
|  | 5 - most deprived | 7367(14.3) | 1.29(1.23-1.36, p<0.001) | - |
| Frailty | Fit | 29010(56.1) | - | - |
|  | Mild frailty | 11648(22.5) | 1.55(1.49-1.61, p<0.001 | - |
|  | Moderate frailty | 6385(12.4) | 2.19(2.10-2.29, p<0.001) | - |
|  | Severe frailty | 4655(9.0) | 3.34(3.20-3.49, p<0.001) | - |
| Side | Right colon | 30916(59.8) | - | - |
|  | Left colon | 20782(40.2) | 0.84(0.82-0.87, p<0.001) | - |
| Total nodes excised | Mean (SD) | 20.9(9.9) | 0.99 (0.99-0.99, p<0.001) | 0.98(0.98-0.99, p<0.001) |
| Number of positive nodes | Mean (SD) | 1.3(2.7) | 1.07(1.06-1.07,p <0.001) | 1.07(1.07-1.08,p<0.001) |

Supplementary table 2 – Cox proportional hazards modelling for the SEER cohort for patients with lymph node metastises only
